# Iowa stream nitrate and the Gulf of Mexico

**DOI:** 10.1371/journal.pone.0195930

**Published:** 2018-04-12

**Authors:** Christopher S. Jones, Jacob K. Nielsen, Keith E. Schilling, Larry J. Weber

**Affiliations:** 1 IIHR-Hydroscience and Engineering, University of Iowa, Iowa City, Iowa, United States of America; 2 Iowa Geological Survey, Iowa City, Iowa, United States of America; Beijing Normal University, CHINA

## Abstract

The main objective of this work was to quantify and update the U.S. Midwest agricultural state of Iowa’s contribution of nitrate-nitrogen to the Mississippi River stream network against the backdrop of the ongoing problem of Gulf of Mexico hypoxia. To achieve this objective, we used stream nitrate and discharge data collected from 1999 until 2016 at 23 Iowa stream sites near watershed outlets, along with publicly-available data for sites downstream of Iowa on the Missouri and Mississippi Rivers. Our analysis shows that Iowa contributes between 11 and 52% of the long-term nitrate load to the Mississippi-Atchafalaya Basin, 20 to 63% to the Upper Mississippi River Basin, and 20 to 89% to the Missouri River Basin, with averages of 29, 45 and 55% respectively. Since 1999, nitrate loads in the Iowa-inclusive basins have increased and these increases do not appear to be driven by changes in discharge and cropping intensity unique to Iowa. The 5-year running annual average of Iowa nitrate loading has been above the 2003 level for ten consecutive years, implying that Gulf hypoxic areal goals, also based on a 5-year running annual average, will be very difficult to achieve if nitrate retention cannot be improved in Iowa. An opportunity exists for land managers, policy makers and conservationists to manifest a positive effect on water quality by targeting and implementing nitrate reducing-practices in areas like Iowa while avoiding areas that are less likely to affect Gulf of Mexico hypoxia.

## Introduction

Coastal Gulf of Mexico eutrophication driven by nutrient enrichment from the Mississippi and Atchafalaya Rivers has been observed and documented since at least 1974 [1,2]. Waters off the coast of Louisiana become degraded as macroalgae and phytoplankton exploit nutrient-rich water and bacterial consumption of their remains consumes dissolved oxygen (DO) [3]. As a result, marine food webs are altered [4], mobile species flee [5,6] and immobile species perish [7] in areas where DO levels drop below 2 mg L^-1^ (hypoxic/hypoxia areas). Economic consequences include decline of commercial fishing catches and recruitment failure of valuable species [8].

In 2001 the Mississippi River/Gulf of Mexico Watershed Nutrient Task Force, a consortium of tribes and federal and state agencies, issued an Action Plan [9] to serve as a strategy for hypoxic area reduction. The group’s long-term goal was to reduce the Gulf area where DO < 2 mg L^-1^ to 5000 km^2^ by 2015. A revised plan was created in 2008, and 12 US states draining to the Mississippi-Atchafalaya River Basin (MARB) continue to implement the 2008 plan. As of 2017, the 5-year running annual average size of the hypoxic area had remained mostly unchanged since 1994, and the Task Force extended the goal target date to 2035 [9].

Although phosphorous, silica, and physical factors contribute, Gulf hypoxia is largely driven by nitrogen loads, mainly nitrate-nitrogen (NO3-N) delivered by the Mississippi and its Atchafalaya distributary [10,11]. The primary source of this NO3-N is row crop agriculture from the U.S. Cornbelt [12,13]. The 2001 Action Plan estimated that a 30% reduction in nitrogen loads would be necessary to reach areal goals; subsequent research demonstrated that 45% reductions were likely needed [14,15,16]. Because NO3-N delivery to streams is mainly from widely scattered non-point sources such as shallow groundwater and farm field drainage lines [12,17], regulations governing its release to the stream network are few. As such, NO3-N load reductions have been dependent upon the voluntary implementation of best management practices (BMPs) by farmers in the MARB [16]. Thus far, documenting NO3-N load reductions linked to policy independent of weather fluctuations has been difficult [18].

The western Cornbelt state of Iowa is a large producer of corn (*Zea mays* L.) and soybeans (*Glycine max* [L.] Merr.) and frequently tops all other U.S. states in the harvested totals of each of these crops [19]. The state is also the leading producer of eggs and pork and the fourth largest producer of feeder cattle [19]. Approximately 90% of the state’s stream NO3-N can be sourced to the 72% of the state’s land area that is in crop cultivation [20]. Previous research in Iowa has shown that a watershed’s NO3-N load is directly linked to the area portion cultivated for corn and soybeans [21,22]. This intense production of carbohydrates and protein has resulted in the state being a leading contributor to MARB NO3-N loads and Gulf hypoxia [23].

Previous researchers have estimated Iowa’s contribution to MARB loading. The Iowa Nutrient Reduction Strategy (INRS) [20] stated Iowa’s average NO3-N contribution to be 280,000 Mg yr^-1^, approximately 29% of the MARB load calculated by Turner and Rabalais [24]. Goolsby et al. [25] used multiple regression models to estimate the combined average load from Iowa and the neighboring state of Illinois to be 35 percent of the total entering the Gulf of Mexico from the MARB. Libra [26] used water monitoring data from the 1990s to report annual Iowa NO3-N loads ranging from 200,000 to 230,000 Mg (25% of MARB total). More recently, Jones et al. [22] estimated Iowa’s 2016 stream NO3-N load to be 477,000 Mg, equivalent to 41 percent of that delivered to the Gulf.

Although some recent research has quantified loading trends within a few of Iowa’s larger interior river basins, e.g. Sprague et al. [18] and Jones et al. [27], and several papers have evaluated concentration trends [28,29,30,31], to our knowledge there have been no recent efforts to quantify trends of Iowa’s statewide contribution to MARB NO3-N loads and Gulf of Mexico Hypoxia. Since strategy and policy development designed to achieve the Mississippi River/Gulf of Mexico Watershed Nutrient Task Force’s objectives are occurring at the state level (i.e. INRS) [20], assessment of statewide NO3-N loading using empirical water quality and quantity data is critical to assess the effectiveness of and bring accountability to these efforts. Thus our research objective was to use long-term (1999–2016) NO3-N concentration and discharge measurements from 23 Iowa stream sites to evaluate loading trends and Iowa’s contribution to MARB loads and Gulf of Mexico Hypoxia in an effort to quantify the effectiveness of the INRS and inform future policy initiatives targeting water quality improvements at the state and regional scales. As part of that, we also quantified Iowa’s long-term contribution to the Upper Mississippi (UMRB) and for the first time to our knowledge, the Missouri River Basin (MoRB).

## Methods

### Study area

**Fig 1:**
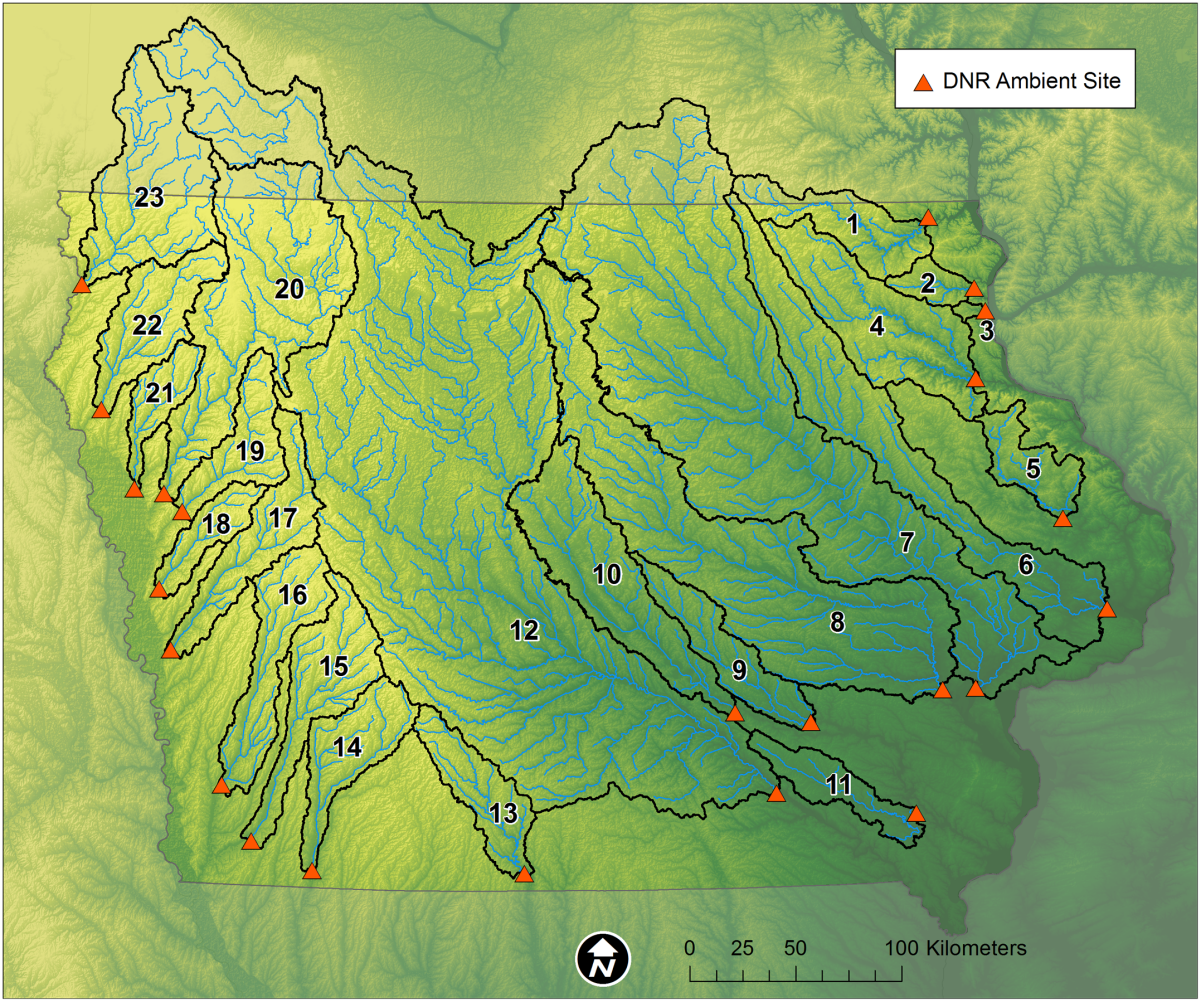
Iowa stream sites and watersheds evaluated in this study. The red triangle indicates the sample location. Numbers correspond to those listed in Table 1.

A total of 23 Iowa watersheds was assessed. These sites are shown in Fig 1 and listed in Table 1. In aggregate they cover 79.8% of Iowa’s area and range in size from 89 (Bloody Run Creek) to 34,751 km^2^ (Des Moines River). Twelve drain to the Upper Mississippi River and 11 to the Missouri River. All the major landforms of Iowa [32] were represented.

**Table 1:** Iowa DNR ambient monitoring sites used in this study.

Watershed Number*	Discharge Gauge Drainage Area (km^2^)	Iowa DNR Site ID	Nitrate Monitoring Site Drainage Area (km^2^)	Latitude	Longitude	Basin	Monitoring Period (WY**)	Fraction of Iowa’s Total Area	Average Row Crop Fraction	Average Annual Discharge (mm)	Average Annual NO3-N yield (kg ha^-1^)	Receiving Stream
1	1,994	10030001	1,987	43.4211	91.5086	Upper Iowa River near Dorchester	1999–2016	0.014	0.544	318	20.1	Mississippi
2	572	10030002	566	43.1119	91.2650	Yellow River near Volney	2005–2016	0.004	0.495	328	22.1	Mississippi
3	88	10220003	89	43.0408	91.2064	Bloody Run Creek near Marquette	1999–2016	0.001	0.475	244	15.5	Mississippi
4	4,002	10220001	4,023	42.7400	91.2617	Turkey River near Garber	2000–2016	0.028	0.570	300	21.6	Mississippi
5	1,308	10490001	1,528	42.1644	90.7294	North Fork Maquoketa River near Hurstville	1999–2016	0.010	0.534	317	23.3	Mississippi
6	6,050	10820001	6,045	41.7669	90.5347	Wapsipinicon River at De Witt	1999–2016	0.041	0.717	340	22.4	Mississippi
7	20,168	10700001	20,159	41.4092	91.2903	Cedar River near Conesville	2000–2016	0.138	0.724	308	21.1	Mississippi
8	11,119	10580002	11,101	41.4239	91.4786	Iowa River near Lone Tree	2007–2016	0.076	0.646	379	22.1	Mississippi
9	1,891	10540001	1,646	41.3008	92.2044	North Skunk River near Sigourney	1999–2016	0.011	0.613	300	18.0	Mississippi
10	4,235	10620001	4,247	41.3558	92.6572	South Skunk River near Oskaloosa	2000–2016	0.029	0.708	295	21.4	Mississippi
11	1,373	10440001	1,379	40.9253	91.6742	Cedar Creek near Oakland Mills	1999–2016	0.009	0.614	301	15.1	Mississippi
12	34,639	10900002	34,751	41.0108	92.4111	Des Moines River Downstream of Ottumwa	2001–2014	0.238	0.651	234	13.9	Mississippi
13	1,816	10270001	1,801	41.6403	93.8081	Thompson Fork—Grand River at Davis City	2000–2016	0.012	0.291	233	4.0	Missouri
14	1,974	10730001	2,046	40.7433	95.0142	West Nodaway River near Shambaugh	2000–2016	0.014	0.537	246	12.6	Missouri
15	2,315	10360001	2,645	41.0086	95.2414	East Nishnabotna River near Shenandoah	1999–2016	0.018	0.689	258	16.6	Missouri
16	1,577	10650001	2,508	41.3900	95.3714	West Nishnabotna River near Malvern	2000–2016	0.017	0.771	234	18.3	Missouri
17	2,256	10430001	2,357	41.6417	95.7822	Boyer River near Missouri Valley	2000–2016	0.016	0.717	203	17.4	Missouri
18	1,054	10430002	1,058	41.8306	95.9311	Soldier River near Pisgah	1999–2016	0.007	0.723	178	12.8	Missouri
19	1,733	10670002	1,668	42.1569	95.8097	Maple River near Mapleton	2000–2016	0.011	0.815	196	19.8	Missouri
20	6,475	10970001	6,958	42.4822	95.7925	Little Sioux River near Smithland	2000–2016	0.048	0.742	186	13.4	Missouri
21	1,044	10970002	1,042	42.2269	96.0778	West Fork Ditch at Hornick	2001–2016	0.007	0.805	166	17.8	Missouri
22	2,295	10750001	2,295	42.5767	96.3111	Floyd River near Sioux City	1999–2016	0.016	0.826	148	18.5	Missouri
23	4,123	10840001	4,351	43.2144	96.2942	Rock River near Hawarden	2000–2016	0.030	0.791	152	14.2	Missouri

*Watershed number corresponds to Fig 1

**WY: Water year, 1 Oct to 30 Sep

### Crop areas

Areas cropped to corn and soybean in the U.S. Midwest (North Dakota-ND, South Dakota-SD, Minnesota-MN, Iowa-IA, Nebraska-NE, Kansas-KS, Missouri-MO, Wisconsin-WI, Illinois-IL, Indiana-IN, and Ohio-OH) were obtained from USDA [19]. These data were evaluated to provide insights into why Iowa NO3-N loading may or may not have changed relative to the Missouri and Mississippi River Basin scales.

### Stream discharge and water yield

**Fig 2:**
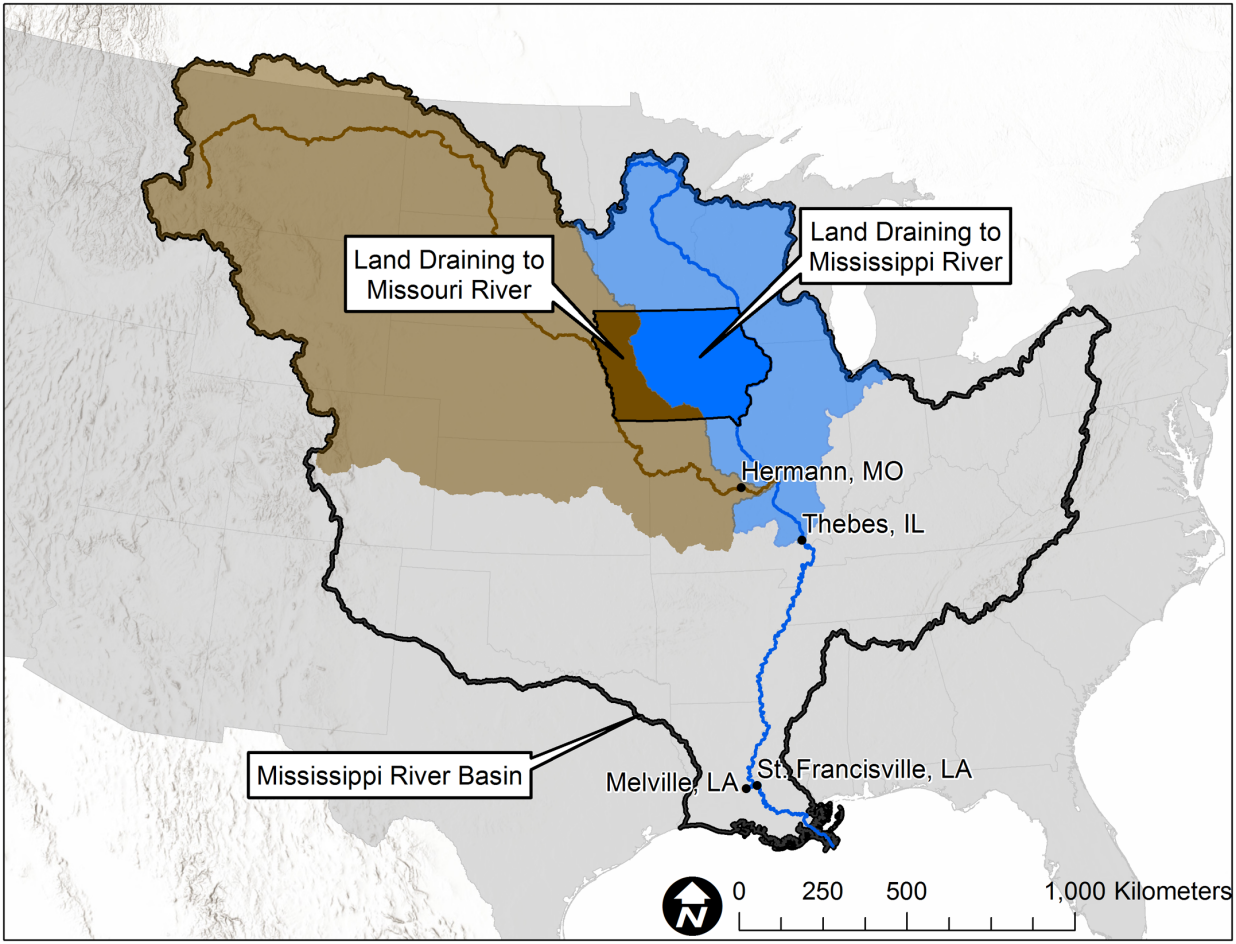
Areas of Iowa draining to the Missouri River and Mississippi River but not the Missouri River.

Daily discharge measurements for the Iowa streams were obtained from the U.S. Geological Survey (USGS) [33]. For comparison purposes, annual (water years 1999–2016, i.e. 1 Oct through 30 Sep) water yield for the Iowa watersheds was determined by summing the daily discharge values and dividing by watershed area. For some of these watersheds, the discharge gauge was not exactly co-located with the NO3-N sampling location. In these circumstances, discharge and water yield were estimated by extrapolating water yield at the actual discharge site to the area draining to the nitrate sampling site. Aggregated discharge and water yield for Iowa in total and areas of the state draining to the Missouri and the Mississippi but not the Missouri were calculated by area-weighting the watershed data available for each individual year. An average water yield (mm) was obtained and then extrapolated to the larger basin area (i.e. Iowa, Iowa areas draining to the Missouri, and Iowa areas draining to the Mississippi but not the Missouri, Fig 2) to derive the total water volume leaving the delineated areas.

Annual (water year) discharge for the Missouri River at Hermann, MO; the Mississippi River at St. Francisville, LA and Thebes, IL, and the Atchafalaya River at Melville, LA were obtained from the USGS NAWQA reports [34]. Discharge for the Upper Mississippi River (i.e. areas draining to Thebes but not the Missouri River) was estimated by subtracting the discharge at Hermann from that at Thebes. Again for comparison purposes, annual water yield for these basins was calculated by dividing annual discharge by watershed area.

### Nitrate measurement

All Iowa NO3-N data were collected as part of the Iowa Department of Natural Resources’ Water Quality Monitoring and Assessment program [35]. The program’s purpose is to provide consistent, unbiased information about the condition of Iowa’s surface and groundwater resources so that decisions regarding the development, management, and protection of these resources may be improved. A fixed network of about 60 sites is point-sampled biweekly-to-monthly for a variety of parameters, including NO3-N. We selected a subset of 23 sites based on their location as a watershed terminus near the Mississippi or Missouri Rivers and the presence of a nearby USGS discharge gauge. All samples were collected as grab (point) samples following a USEPA-approved Quality Assurance Project Plan and then were immediately preserved and delivered to the State of Iowa Hygienic Laboratory where they were analyzed using USEPA Method 353.2 [36]. Sample sites and collection and lab procedures were unchanged during the period of study. Because NO3-N moves in soluble form [37], and because fixed-location samples provide a robust proxy for cross-sectional average NO3-N concentrations [38] we assumed that NO3-N was well-mixed within the stream at the sample locations.

Concentration data for non-analysis days were estimated using linear interpolation [37, 39] and daily loads of NO3-N were calculated by multiplying concentration by daily average discharge while annual water year loads were calculated by summing the daily loads. Not all sites were sampled every year. As such, aggregated loads and yields for Iowa and areas of the state draining to the Missouri and the Mississippi but not the Missouri were calculated by area-weighting the watershed data available for each individual year. An average per hectare NO3-N yield (kg ha^-1^) was obtained and then extrapolated to the larger basin area (i.e. Iowa, Iowa areas draining to the Missouri, and Iowa areas draining to the Mississippi but not the Missouri).

Annual (water year) NO3-N loads, 1999–2016, for the Missouri River at Hermann, MO, the Mississippi River at Thebes, IL, and the combined Atchafalaya-Mississippi load were obtained from the USGS NAWQA reports [34]. Loads for the Upper Mississippi River (i.e. areas draining to Thebes but not the Missouri River) were estimated by subtracting the NO3-N load at Thebes from that at Hermann.

## Results

### Crop area

**Fig 3:**
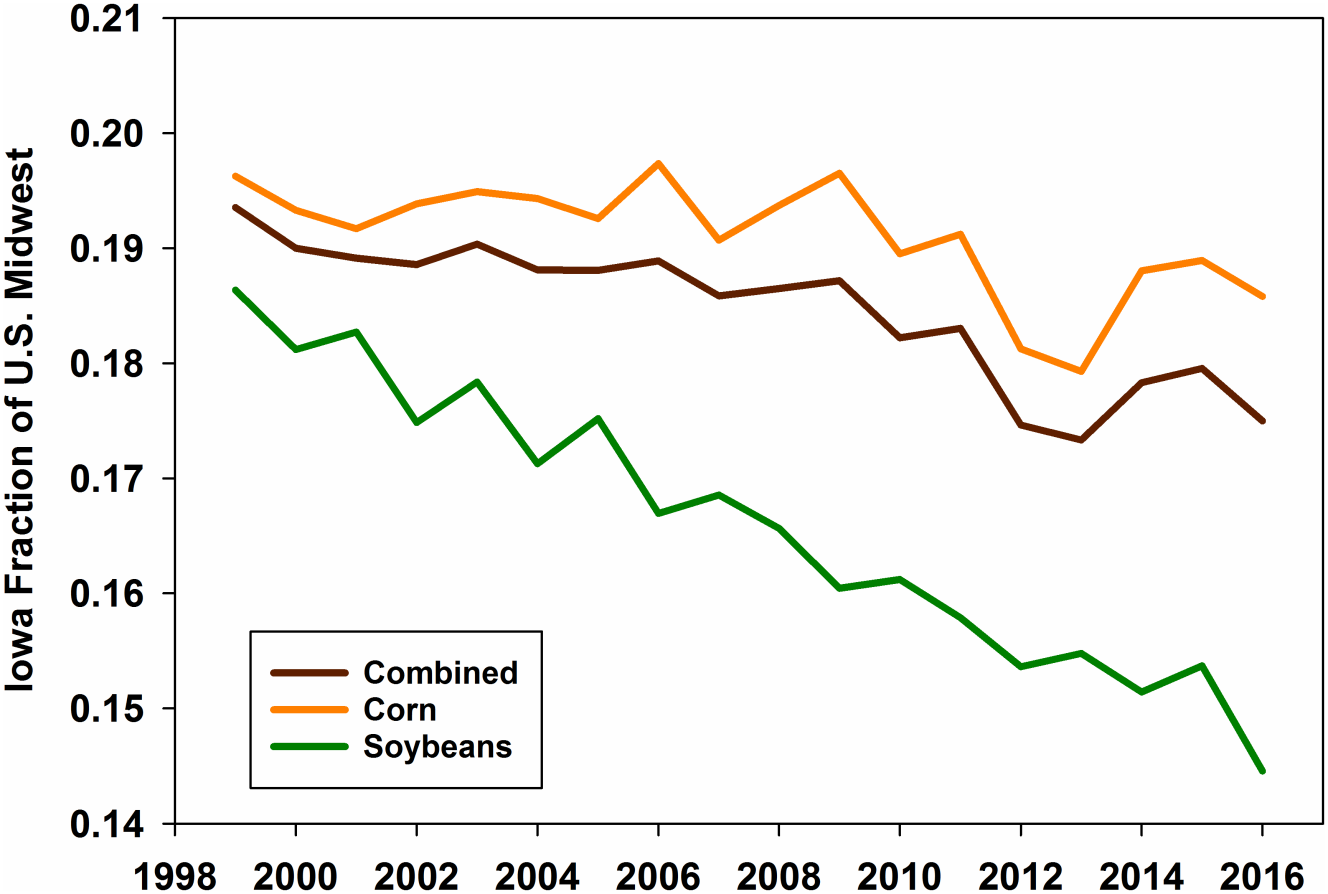
Iowa fraction of combined corn and soybean area from North Dakota, South Dakota, Nebraska, Kansas, Minnesota, Iowa, Missouri, Wisconsin, Illinois, Indiana and Ohio.

When considering the U.S. Cornbelt states draining to the MARB (ND, SD, NE, KS, MN, IA, MO, WI, IL, IN, OH), Iowa had the largest average combined area in corn and soybean production (93,340 km^2^) and the largest average area portion in cultivation for these crops (0.64). The state also had the most total area in corn production in each year of the study and the most area in soybean production in 14 of the 18 years. Overall, however, the state’s share of all corn/soybean area in the region steadily declined (Fig 3) as cropped areas increased in other states, especially MN, ND, SD and KS. Iowa’s decline in the share of the region’s soybean area was especially pronounced, dropping from 18.6% in 1999 to 14.5% in 2016. Total corn-soybean area in Iowa ranged from 91,867 (2001) to 95,307 km^2^ (2014) while these areas ranged from 484,021 (1999) to 568,644 km^2^ (2015) in the Cornbelt region as a whole. Thus while corn-soybean area was increasing approximately 21% across the Cornbelt, this increase was < 4% in Iowa.

### Water yield

**Fig 4:**
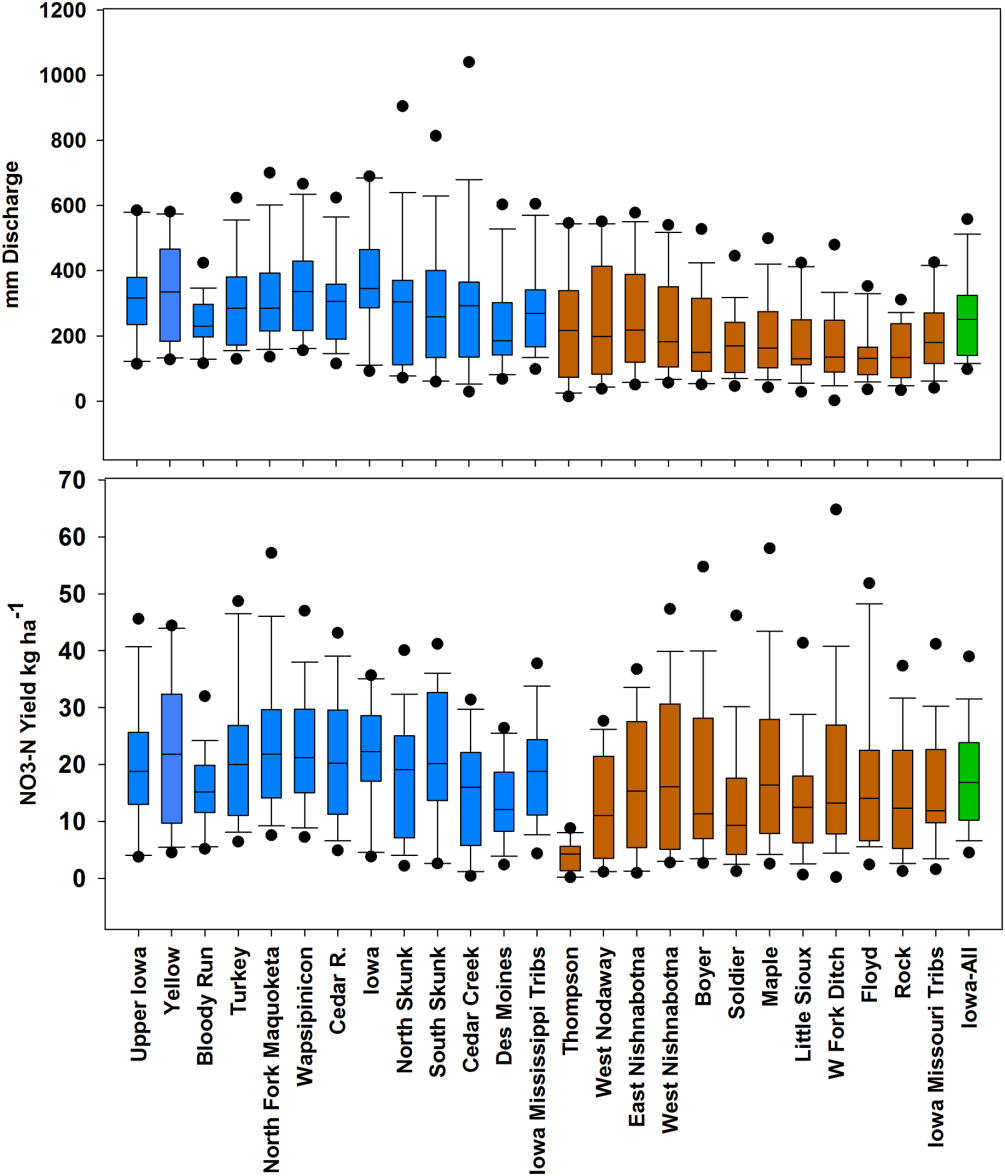
Box plots of water yield (top) and NO3-N yield (bottom) for the period of study. Streams draining to the Mississippi but not the Missouri are shown in blue while streams draining to the Missouri are shown in brown. The overall Iowa averages are shown in green. The boxes bracket the 25th-75th percentiles; the line in the box indicates the median; the whiskers the 10th and 90th percentiles, and the dots are data points less than (greater than) the 10th (90th) percentiles.

Annual water yield across Iowa varied from 98 mm in the drought year of 2012 to 605 mm in 2016 with an overall average of 264 mm. Water yield averaged 199 mm in the first half of the record (1999–2007) and 328 mm the second half (2008–2016). The largest annual water yield from any Iowa watershed was 1040 mm for the South Skunk River in 2010. Water yield from Iowa areas draining directly to the Mississippi River was 45% higher than areas draining to the Missouri (289 versus 199 mm). In the larger receiving basins, average water yield ranged from 57 mm (MoRB) to 203 (MARB) to 307 (UMRB). The largest annual water yield for all three of these basins occurred in 2010; likewise the lowest water yield year for all three occurred in the same year—2006. Water yield values for all the Iowa watersheds are shown in Table 1 and Fig 4.

### Nitrate loads and yields

Annual NO3-N yield from Iowa (Fig 4) ranged from 4.5 (2012) to 38.8 kg ha^-1^ (2016), translating into NO3-N loads of 66,000 to 567,000 Mg. Annual yields to the Missouri River ranged from 1.6 (2000) to 41.2 kg ha^-1^ (2016), equivalent to loads of 7100 to 186,000 Mg. Yields from Iowa areas draining directly to the Upper Mississippi River varied from 4.3 (2012) to 37.7 kg ha^-1^ (2016), translating to loads of 44,000 to 379,000 Mg. Between watersheds, average yields for the entire period ranged from 4 kg ha^-1^ (Thompson Fork) to 23.3 kg ha^-1^ (North Fork of the Maquoketa). The largest one-year yield was 64.4 kg ha^-1^ in the West Fork Ditch (2016).

**Fig 5:**
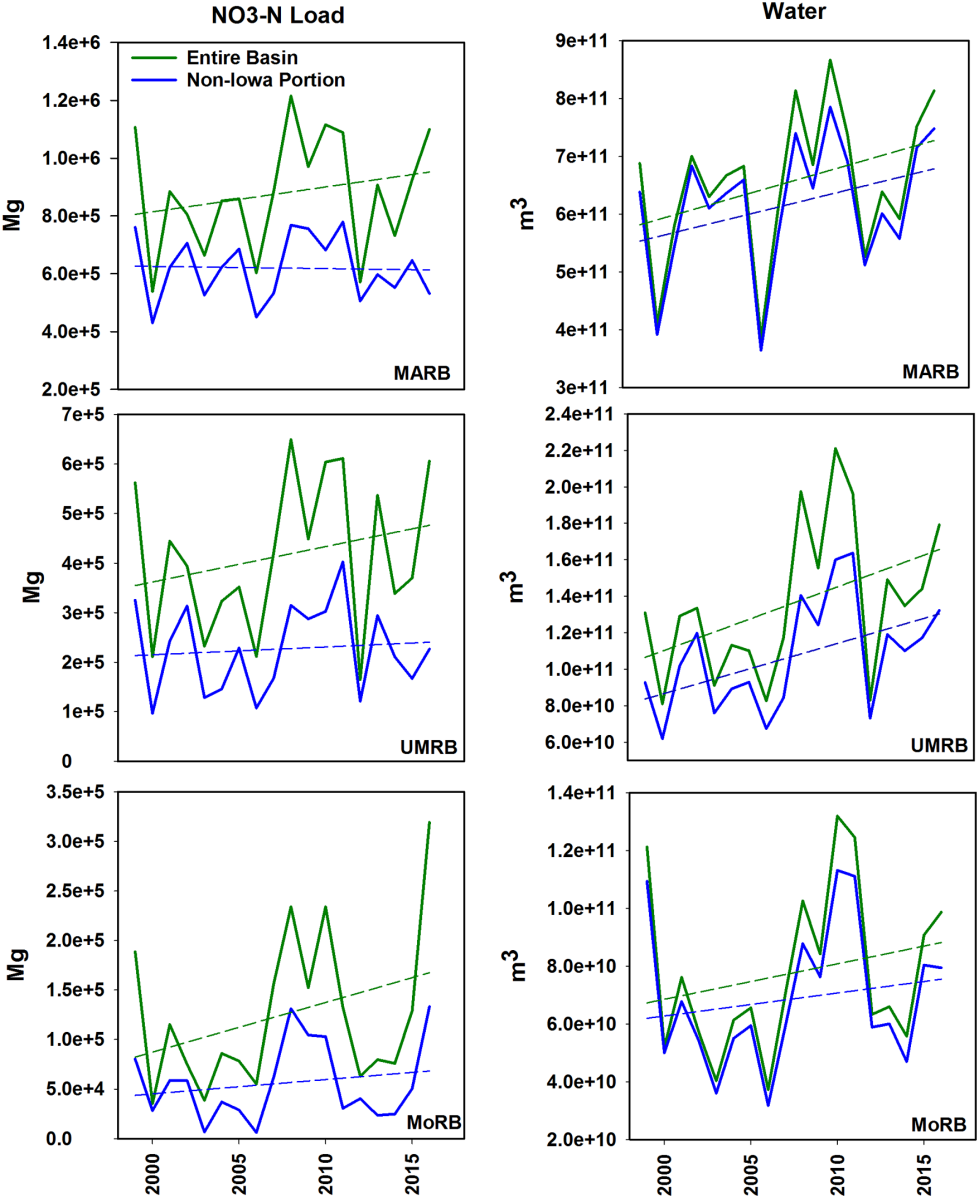
Loads of NO3-N (left) and total discharge (right) for the Mississippi-Atchafalaya River Basin (MARB), Upper Mississippi River Basin (UMRB) and Missouri River Basin (MoRB). The green lines indicate the entire basin; the blue lines indicate the non-Iowa portions.

In the larger receiving basins annual loads ranged from 539,000 to 1,216,000 Mg (MARB), 21,000 to 650,000 Mg (UMRB), and 35,000 to 319,000 Mg (MoRB) (Fig 5). Meanwhile yields varied from 1.8 to 4.1 kg ha^-1^ (MARB), 3.7 to 14.6 kg ha^-1^ (UMRB), and 0.3 to 2.4 kg ha^-1^ (MoRB). The lowest yields occurred in 2000 for the MoRB and MARB and 2012 for the UMRB. The highest yields occurred in 2016 (MoRB) and 2008 (MARB and UMRB). Comparing variations in annual yield, Iowa in aggregate varied over a factor of 8.6; the MoRB 8.0; UMRB, 3.9; and the MARB, 2.3. Annual yields for the West Fork Ditch in Iowa varied over a factor of 340.

**Fig 6:**
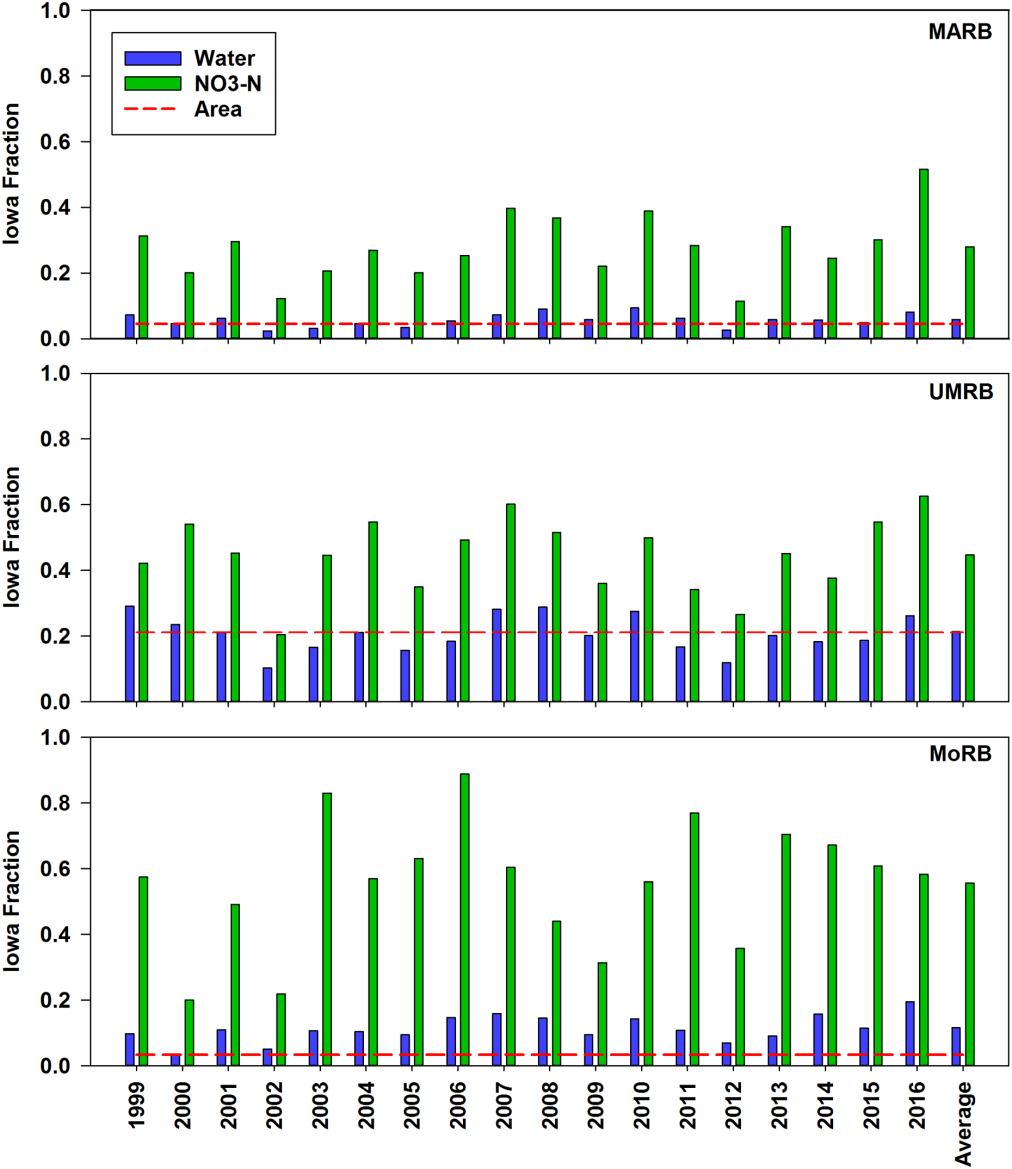
Iowa portion of the total discharge (blue bars), NO3-N load (green bars) and land area (red line) in the Mississippi-Atchafalaya River Basin (MARB), Upper Mississippi River Basin (UMRB) and Missouri River Basin (MoRB).

The Iowa portion of the MARB, UMRB and MoRB load is shown in Fig 6. Iowa’s NO3-N load portion in the MARB ranged from 11 (2012) to 52% (2016) and averaged 29% for the 18 year period. Iowa areas draining directly to the Upper Mississippi River contributed 20 (2002) to 63% (2016) of the UMRB load with an average of 45%. Meanwhile Iowa watersheds draining to the Missouri River delivered 20 (2000) to 89% (2006) of the total MoRB load measured at Hermann, MO, averaging 55% over the 18-year period.

Using total discharge and total NO3-N load for the period (1999–2016), flow-weighted average (FWA) NO3-N concentrations were calculated for Iowa, the MARB, UMRB, and MoRB basins and the non-Iowa portions of these basins. These are shown in Table 2. Iowa contributions of water and NO3-N nearly double the FWA concentration of the Missouri River; likewise Iowa contributions raise the UMRB and MARB FWA concentration 44% and 33% respectively. Also shown in Table 2 are the FWA concentrations in the MARB, UMRB and MoRB if Iowa’s total NO3-N load was reduced 45%, the goal for the state set by the INRS. In this circumstance, FWA concentrations would decline 15, 26, and 33% for the MARB, UMRB, and MoRB, respectively. These concentrations assume average discharge from 1999–2016 would remain unchanged in future years.

**Table 2:** Flow-weighted average NO3-N concentrations (mg L^-1^) in the Mississippi-Atchafalaya River Basin (MARB), Upper Mississippi River Basin (UMRB) and Missouri River Basin (MoRB), the Iowa and non-Iowa portions of each of those basins, and the concentration of the entire basins if the Iowa Nutrient Reduction Strategy goal of 45% load reduction was met.

Basin	Entire Watershed	Iowa Portion	Non-Iowa Portion	Entire Basin if Iowa load declines 45%
MARB	1.34	6.74	1.01	1.16
UMRB	3.05	6.50	2.12	2.43
MoRB	1.61	7.64	0.82	1.21

## Discussion

Iowa’s 18-year average NO3-N load contribution to the MARB was 29% of the total, consistent with some previous estimates, especially that of the INRS [20] which also estimated a 29% contribution. Libra’s 1998 estimate [26] of 25% is somewhat less than our calculated amount, while Goolsby’s 2000 estimate [23] of 35% for Iowa and the very similar neighboring state of Illinois seems likely to be low. We should note that these other estimates were made 20 or more years ago, near the beginning of our period of record. Recently Jones et al. [22] used a high-frequency sensor network measuring NO3-N in 13 major Iowa basins to estimate the 2016 calendar year load for the state to be 477,000 Mg and 41% of the MARB load, less than the 568,000 Mg (52%) calculated here for the 2016 water year. It should be emphasized that December 2015 was exceptionally wet in Iowa with a large NO3-N load, which would have figured into the 2016 water year but not the 2016 calendar year. In any case, the Iowa portion for both the 2016 calendar and water years was very high and implies that Iowa can be a strong driver Gulf of Mexico hypoxia.

As our data was part of the IDNR ambient monitoring program, it was generated from point measurements and did not include storm event samples. This is consistent with the other previous estimations (i.e. [20, 25, 26]) of Iowa NO3-N loading cited here. We believe deliberate inclusion of storm event samples is not likely to alter our calculated load totals, as up to 80% of the NO3-N load in Iowa occurs during baseflow [40] and that it is well established that weekly and biweekly grab samples are adequate for quantifying NO3-N loss at the landscape scale [18, 41, 42]. Lee et al. [37] evaluated several methods and sampling strategies for determining decadal NO3-N loads. In that study, linear interpolation of point data from a “uniform” sampling protocol (like that conducted here) produced a mean percent error and root mean squared percent error of -2 and 4 and respectively, only slightly different from errors produced by high flow sampling (1 and 3% respectively). For these reasons, we believe the data from the ambient monitoring program is adequate for quantifying Iowa NO3-N loads.

We are not aware of other detailed estimates of Iowa’s NO3-N load to the MoRB and UMRB. It’s clear that Iowa is a major contributor to both, especially the MoRB. In some years, the Missouri River would have nearly no NO3-N without contributions from Iowa (e.g. 2003, 2006, 2011) (Fig 6). Because of lower average precipitation, Libra [26] estimated loading from Missouri River tributaries in western Iowa to be lower than the Iowa tributaries draining to the east toward the Mississippi, and our analysis confirms this conclusion. To our knowledge, however, how western Iowa streams draining only 3% of the Missouri Basin can dominate overall Missouri River NO3-N loading has not been previously reported in any published literature. This illustrates the importance of implementing NO3-N mitigation strategies that address not only the level, tile-drained landscapes in northern and eastern Iowa but also the hillier terrain of western Iowa where constructed drainage is less common. Iowa is also a strong contributor to the UMRB NO3-N load, with an overall portion of 45% for the period of record. Similar to the MoRB, we are not aware of detailed estimates of Iowa’s proportional NO3-N load contribution solely within the UMRB.

For all three major basins, Iowa’s disproportionate load contribution is not consistent with its contribution of water. In the MARB, the state contributes 5.9% of the water and 29% of the NO3-N while occupying 4.5% of the basin area; for the UMRB, 21% of the water and 45% of the NO3-N with 21% of the land area; and for the MoRB, 12% of the water and 55% of the NO3-N but only 3.3% of the watershed area (Fig 6). This and related FWA concentrations (Table 2) indicate that the supply of loss-vulnerable NO3-N on the landscape is much higher in Iowa than in the rest of the larger basins. Certainly a factor contributing to Iowa’s disproportionate NO3-N contribution is the magnitude of land area committed to crop production. The state has the largest areas in corn and soybean production and the largest fractions of total area in production of the Cornbelt states, an important driver of watershed NO3-N loading [20].

We illustrate both basin-wide NO3-N load and water discharge in the MARB, UMRB, and MoRB and these same parameters in the non-Iowa portions of these watersheds in Fig 5. Regression lines highlight how the paired basins (Iowa-inclusive and non-Iowa portion) compared with respect to NO3-N loading and discharge. Although the differences between the regression lines were not statistically significant due to large year-to-year variations, the lines nonetheless illustrate how the paired basins have behaved somewhat differently for NO3-N loading, and similarly for discharge. While NO3-N loads appear relatively unchanged in the non-Iowa portions of the MARB, UMRB, and MoRB, inclusion of Iowa increases the slope of a regressed line of basin loads (Fig 5). Since NO3-N loads are highly dependent upon discharge [18], differences between NO3-N load trends and discharge trends would therefore imply differences in NO3-N supply on the landscape and NO3-N concentration in the studied streams and basins. This implies that changes have occurred in the Iowa landscape (besides increased discharge) that are increasing NO3-N loads, or that changes are occurring in the non-Iowa areas of the MARB, UMRB and MoRB, but not in Iowa, that are preventing increases in NO3-N loading. Interestingly, areas cropped to corn and soybean have not increased much in Iowa compared to the rest of the Cornbelt (Fig 3), so any landscape changes that are driving changes in NO3-N loads would necessarily be due to crop/field management, weather patterns, or possibly legacy NO3-N [43], in that much of Iowa’s land area has been committed to corn and soybean production for many decades. One other possible factor is drainage tile. Although accurate records are sparse to non-existent, much of Iowa’s farmland requires artificial drainage to optimize conditions for corn and soybean production. There is anecdotal evidence [44] that improvements in Iowa’s drainage infrastructure have been extensive in recent years. Since this is the primary NO3-N delivery mechanism for Iowa streams, it would seem reasonable that this could be affecting NO3-N loads in Iowa more than other states where constructed drainage is less common.

**Fig 7:**
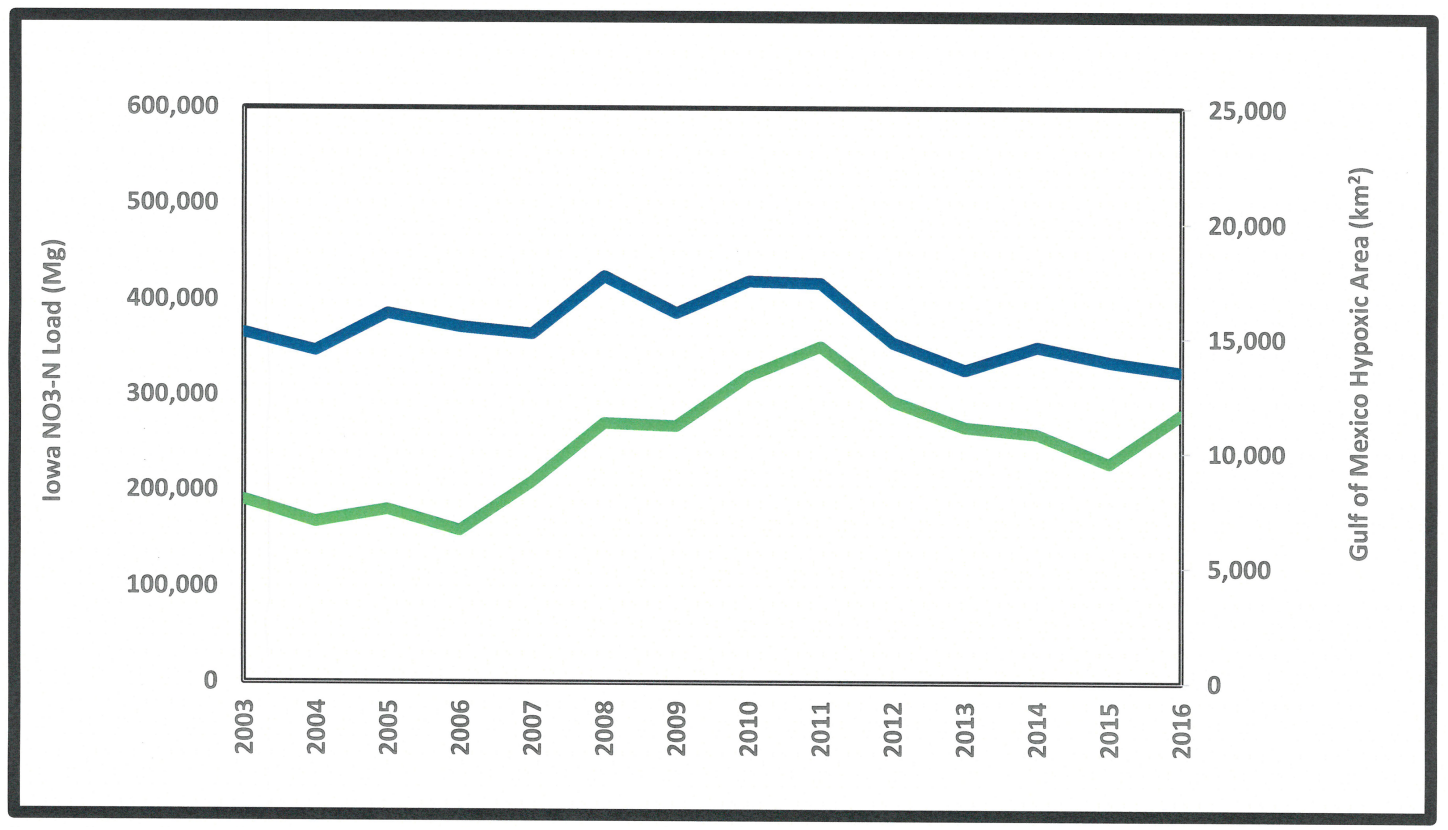
Five-year running annual average of Gulf of Mexico hypoxic area (blue) and Iowa stream NO3-N loads (green).

Since climate is a contributor to the extent of Gulf of Mexico hypoxia [45], the Gulf Hypoxia Task Force’s goal for area reduction is based on a 5-year moving average that presumably accounts for year-to-year weather variations that could be expected to be large, especially in the mid-continental area of the MARB that includes Iowa. Fig 7 shows the 5-year moving average of Gulf Hypoxia area [46] and Iowa loads calculated for the period of record here. Since 2003 (the first year of our water quality record where the 5-year moving average can be calculated), the value for the hypoxic area has been far larger than the Task Force’s goal (5000 km^2^), although the current value is slightly smaller than in 2003. The Iowa 5-year moving average NO3-N load value, however, is about 40% higher than 2003 and has been higher than the 2003 value for the last ten consecutive years. With the role of NO3-N as a strong driver of Gulf hypoxia [10,11], focus on reducing loss of this pollutant from its primary source areas like Iowa is crucial. With the state responsible for as much as half of the MARB NO3-N load, conservation practices such as cover crops [47], constructed wetlands [48] and restored oxbows [49,50] would seem to have the greatest potential to affect Gulf hypoxia if implemented in this area of large NO3-N loss.

## Conclusions

Iowa’s NO3-N load contribution to the MARB, UMRB, and MoRB averaged 29, 45, and 55% respectively for the water year period 1999–2016, and can be as high as 52, 63 and 89%, respectively. When considering these basins, NO3-N loading from the non-Iowa portions seems to be stable or increasing at a slower rate than the Iowa-inclusive area while discharge is behaving similarly between the non-Iowa and Iowa-inclusive areas. This implies that the dynamics of weather and discharge are not primarily responsible for differences in the NO3-N patterns that exist between Iowa and the rest of the MARB, UMRB, and MoRB since 1999. These NO3-N patterns are occurring against a backdrop of slow expansion (< 4%) in Iowa crop area but much larger expansion in Cornbelt crop areas (21%). Data reported here indicates that if Iowa can reach its 45% load reduction goal, FWA NO3-N concentrations would decline 15, 26, and 33% for the MARB, UMRB, and MoRB, respectively. Land managers, policy makers and conservationists should view this as an opportunity to implement NO3-N reducing practices in areas such as Iowa where they are likely to produce measurable improvements in Missouri and Mississippi River nitrate loads.

## Supporting information

S1 Dataset Raw nitrate concentration and discharge data for the stations used in this study. (Data A) Upper Iowa River. (Data B) Yellow River. (Data C) Bloody Run Creek. (Data D) Turkey River. (Data E) North Fork Maquoketa River. (Data F) Wapsipinicon River. (Data G) Cedar River. (Data H) Iowa River. (Data I) North Skunk River. (Data J) South Skunk River. (Data K) Cedar Creek. (Data L) Des Moines River. (Data M) Thompson River. (Data N) West Nodaway River. (Data O) East Nishnabotna River. (Data P) West Nishnabota River. (Data Q) Boyer River. (Data R) Soldier River. (Data S) Maple River. (Data T) Little Sioux River. (Data U) West Fork Ditch. (Data V) Floyd River. (Data W) Rock River. (Data X) Atchafalaya River nitrate loads. (Data Y) Atchafalaya River discharge. (Data Z) Missouri River nitrate loads. (Data AA) Missouri River discharge. (Data BB) Mississippi River at Thebes nitrate loads. (Data CC) Mississippi River at Thebes discharge. (Data DD) Mississippi River at St. Francisville nitrate loads. (Data EE) Mississippi River at St. Francisville Discharge.
(XLSX)
